# Neutrophil diversity is associated with T-cell immunity and clinical relevance in patients with thyroid cancer

**DOI:** 10.1038/s41420-024-01970-z

**Published:** 2024-05-08

**Authors:** Seong Eun Lee, Bon Seok Koo, Pureum Sun, Shinae Yi, Na Rae Choi, Jiyeon Yoon, Seon-Young Kim, Seon-Kyu Kim, Seongyeol Park, Aliya Lakhani, Samantha O’Keeffe, Junyoung O. Park, Da Hyun Kang, Yea Eun Kang

**Affiliations:** 1 Research Institute for Medical Sciences, College of Medicine, Chungnam National University, Daejeon, Republic of Korea; 2 Department of Otolaryngology-Head and Neck Surgery, Chungnam National University Hospital & College of Medicine, Daejeon, Republic of Korea; 3 Department of Medical Science, Chungnam National University, Daejeon, Republic of Korea; 4 Korea Research Institute of Bioscience and Biotechnology, Deajeon, Republic of Korea; 5 Stanford Cancer Institute, Stanford University School of Medicine, Stanford, CA USA; 6 Department of Chemical and Biomolecular Engineering, University of California, Los Angeles, Los Angeles, CA 90095 USA; 7 Department of Internal Medicine, Chungnam National University Hospital & College of Medicine, Daejeon, Republic of Korea; 8 Department of Medical Science, Chungnam National University School of Medicine, Daejeon, 35015 Republic of Korea

**Keywords:** Prognostic markers, Translational research

## Abstract

Neutrophil heterogeneity is involved in autoimmune diseases, sepsis, and several cancers. However, the link between neutrophil heterogeneity and T-cell immunity in thyroid cancer is incompletely understood. We investigated the circulating neutrophil heterogeneity in 3 undifferentiated thyroid cancer (UTC), 14 differentiated thyroid cancer (DTC) (4 Stage IV, 10 Stage I–II), and healthy controls (*n* = 10) by transcriptomic data and cytometry. Participants with UTC had a significantly higher proportion of immature high-density neutrophils (HDN) and lower proportion of mature HDN in peripheral blood compared to DTC. The proportion of circulating PD-L1^+^ immature neutrophils were significantly increased in advanced cancer patients. Unsupervised analysis of transcriptomics data from circulating HDN revealed downregulation of innate immune response and T-cell receptor signaling pathway in cancer patients. Moreover, UTC patients revealed the upregulation of glycolytic process and glutamate receptor signaling pathway. Comparative analysis across tumor types and stages revealed the downregulation of various T-cell-related pathways, such as T-cell receptor signaling pathway and T-cell proliferation in advanced cancer patients. Moreover, the proportions of CD8+ and CD4+ T effector memory CD45RA+ (TEMRA) cells from peripheral blood were significantly decreased in UTC patients compared to DTC patients. Finally, we demonstrated that proportions of tumor-infiltrated neutrophils were increased and related with poor prognosis in advanced thyroid cancer using data from our RNA-seq and TCGA (The Cancer Genome Atlas) data. In conclusion, observed prevalence of circulating immature high-density neutrophils and their immunosuppressive features in undifferentiated thyroid cancers underscore the importance of understanding neutrophil dynamics in the context of tumor progression in thyroid cancer.

## Introduction

Neutrophils participate in defense against bacterial infections and facilitate innate and adaptive immune responses during inflammation. In cancer, neutrophils and proinflammatory immune cells are recruited from the bloodstream in response to the early signs of inflammation [1]. Neutrophils are also recruited to primary tumors by diverse stimuli and have the multifaceted impact on the tumor microenvironment [2]. Neutrophils have essential properties in regulating tumor metastasis and tumorigenesis, and they also have been known to have tumor suppressive roles [3, 4]. Tumor-associated neutrophils (TANs) accelerate tumor growth and metastasis through the production of several growth and inflammatory factors [5, 6]. However, neutrophils also have the opposite role for promoting the anti-tumor functions by enhancing the efficacy of cytotoxic T cells [7].

Recently, several advanced experimental methods have shown that neutrophil heterogeneity is important not only in healthy states but also in diseased states [8–10]. Neutrophil heterogeneity has been revealed through single-cell transcriptomics or advanced flow cytometry, as represented by the presence of tumor-promoting neutrophils and tumor-suppressive neutrophils in several tumor models [11–13]. Circulating neutrophils can also be classified into the commonly known high-density neutrophils (HDN) and low-density neutrophils (LDN) based on their density separation in a discontinuous density gradient [14]. While HDN is the neutrophil that generally occupies the largest proportion, LDN has been found to increase in inflammation status, and many studies have focused on the role of circulating immunosuppressive LDN in disease status, such as HIV infection and sepsis [15, 16]. However, neutrophil heterogeneity, which also plays an immunosuppressive role in HDN, has recently been discovered [14, 17], and the diversity of neutrophils in disease situations is not yet fully known. In thyroid cancer, soluble factors derived from cancer cells was known to promote recruit and activate TANs [18]. Anaplastic thyroid cancer (ATC) conditioned media(CD) induce neutrophil extracellular DNA traps(NET release), whereas differentiated thyroid cancer (DTC) and normal thyroid CM did not [19]. These data suggest the impact of neutrophil in orchestrating the inflammatory response in thyroid cancer. However, to date, the immunoregulatory properties of LDN and HDN in thyroid have not been investigated.

Due to the short life of circulating neutrophils [20], most previous studies have identified the impact of neutrophils by neutrophil-to-lymphocyte ratio (NLR) in the peripheral blood, which correlates with poor prognosis or cancer development in advanced cancers [21]. Especially in thyroid cancer, high baseline NLR were related with poor prognosis and have identified the predictive factor for radioactive iodine therapy [22–25]. NLR is not only known to be increased in undifferentiated cancer compared to differentiated cancer, but can also be used as a prognostic biomarker associated with treatment response to lenvatinib [26, 27]. However, the diagnostic and prognostic significance of NRL is controversial [22, 28] and a deeper understanding of peripheral neutrophils based on an integrated analysis using advanced technologies is necessary.

Here, we investigated circulating neutrophil heterogeneity using flow cytometry and transcriptomics in patients with thyroid cancer. Based on transcriptomics, we identified that immune-related signaling and specific genes were significantly altered in relation to tumor type and prognosis and validated our results using our own bulk RNA sequencing data of tumor samples to identify the impact of neutrophil in tumor progression.

## Results

### High-density neutrophil (HDN) were decreased in thyroid cancer patient

**Fig. 1:**
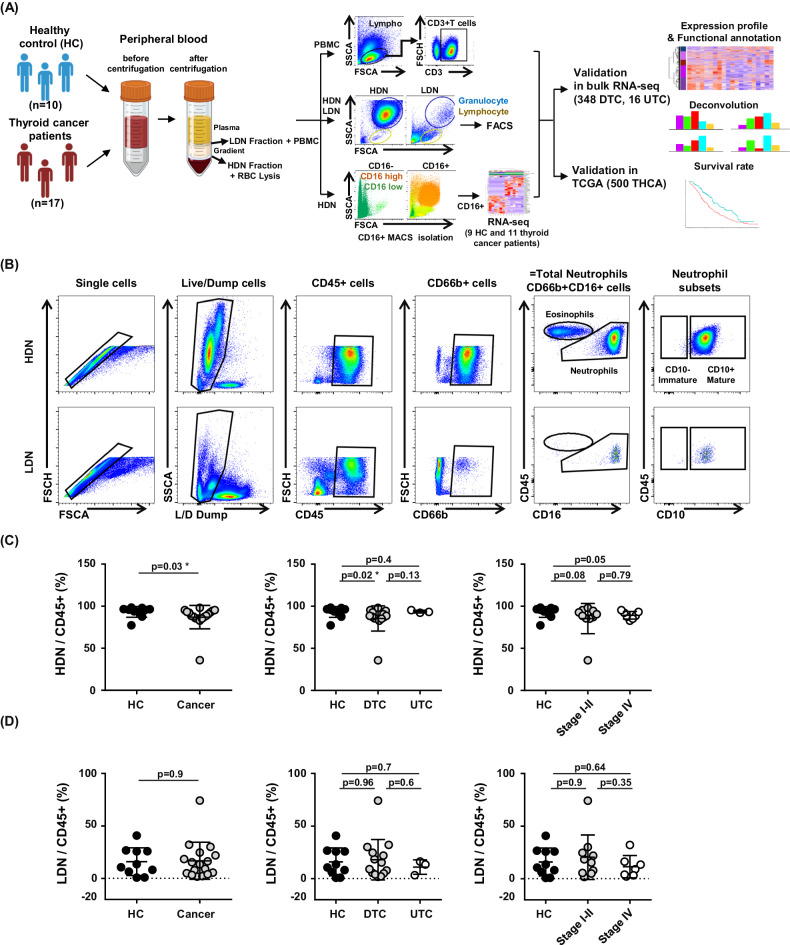
Study scheme and flow cytometry analysis of neutrophil subsets related with gradient in thyroid cancer and healthy controls. **A** Overview of the study scheme. **B** Gating strategies of neutrophils applied in flow cytometry analyses. **C** Frequency of HDN neutrophils in cancer patients (*n* = 17) and healthy controls (*n* = 10). **D** Frequency of LDN neutrophils in cancer patients and healthy controls. Data were expressed as the mean ± SD (HC, *n* = 10; Cancer, *n* = 17; DTC, *n* = 14; UTC, *n* = 3; Stage I–II, *n* = 10; Stage IV, *n* = 7).

We quantified changes in peripheral blood cell populations, including neutrophils and peripheral mononuclear cells, using an isolation protocol in 10 healthy controls and 17 patients with thyroid cancer (Fig. 1A). We performed bulk RNA sequencing of high-density neutrophils (HDN) in 9 controls and 11 patients with thyroid cancer to investigate changes in tumor-specific expression in neutrophils. We performed bulk RNA sequencing from 364 tissues from our previous study [29] for deconvolution analysis to identify the impact of the neutrophil population, and the TCGA database was also analyzed to confirm our findings. First, we identified HDN and LDN populations in relation to tumor type and prognosis. The total neutrophil populations were defined as CD66b+ and CD16+ cells (Fig. 1B). The total neutrophil population in the high-density gradient significantly decreased in patients with thyroid cancer (Fig. 1C). However, there was no difference associated with tumor type or stage. There were no changes in low-density gradient neutrophils between patients with thyroid cancer and controls (Fig. 1D).

### Mature neutrophils were decreased and immature neutrophils are enriched in thyroid cancer

Next, we compared mature and immature neutrophil populations. CD66b+CD16+ cells were defined as neutrophils, and CD10 was used as a marker to distinguish between mature and immature cells; neutrophil gating strategies were applied in flow cytometry analyses. Distinction between CD10+ cells and CD10− cells was based on Fluorescence Minus One (FMO) control (Supplementary Fig. S1).

**Fig. 2:**
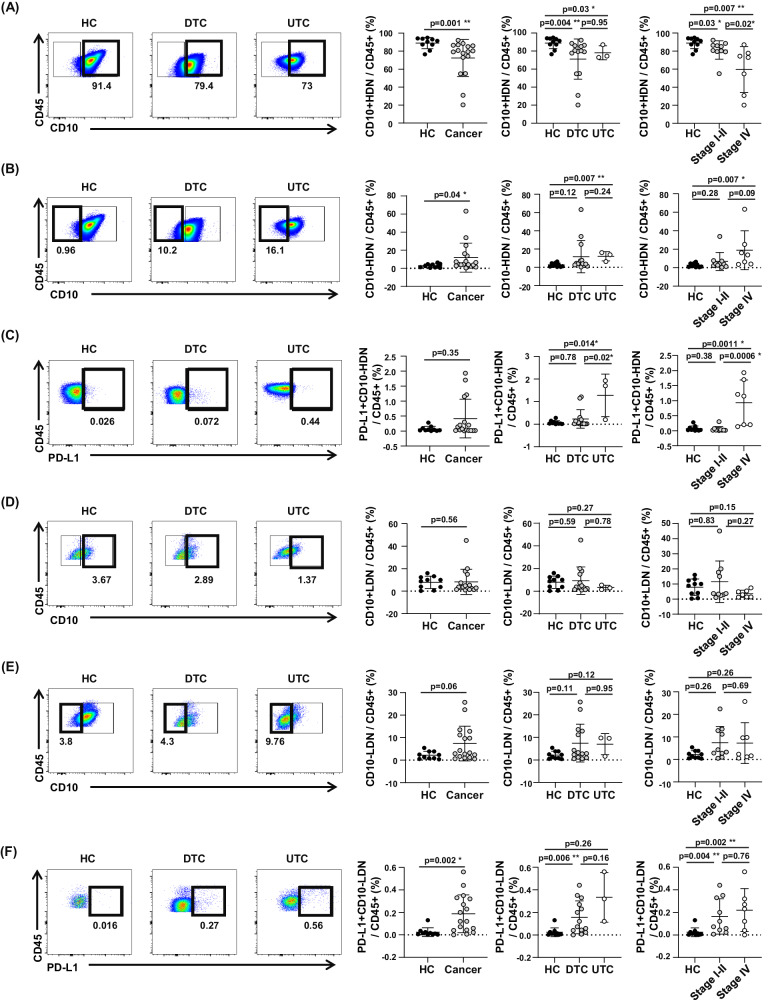
Study scheme and flow cytometry analysis of neutrophil subsets in thyroid cancer and healthy controls. **A** Frequency of CD10+ mature neutrophils in HDN from cancer patients and healthy controls. **B** Frequency of CD10− immature neutrophils in HDN from cancer patients and healthy controls. **C** Frequency of PD-L1+CD10− immature neutrophils in HDN from cancer patients and healthy controls. **D** Frequency of CD10+ mature neutrophils in LDN from cancer patients and healthy controls. **E** Frequency of CD10- immature neutrophils in LDN from cancer patients and healthy controls. **F** Frequency of PD-L1+CD10− immature neutrophils in LDN from cancer patients and healthy controls. Data were expressed as the mean ± SD (HC, *n* = 10; Cancer, *n* = 17; DTC, *n* = 14; UTC, *n* = 3; Stage I–II, *n* = 10; Stage IV, *n* = 7).

The frequency of CD10+ mature neutrophils in HDN was significantly lower in cancer patients (Fig. 2A). We compared the frequency of mature neutrophils concerning the tumor type and stage. The frequency of mature neutrophils in HDN was significantly lower in cancer patients than that in healthy controls (Fig. 2A). In contrast, the frequency of CD10- immature neutrophil in HDN was significantly higher than in healthy controls (Fig. 2B). The number of immature neutrophils in HDN significantly increased in patients with UTC and stage IV thyroid cancer (Fig. 2B). Additionally, the frequency of PD-L1+CD10− immature neutrophils in HDN in UTC was significantly higher than both in healthy controls and DTC (Fig. 2C.) The number of PD-L1+CD10− immature neutrophils was also much higher in patients with stage IV (Fig. 2C). Although the total number of LDN did not change, we investigated the frequency of mature or immature neutrophils at a low density. In UTC and stage IV patients, mature neutrophil LDN, similar to HDN, showed a tendency to decrease, but the difference was not significant. (Fig. 2D). In immature neutrophils, a significant increasing trend was observed in LDN in stage I–III patients compared to that in healthy controls, but no other statistical significance was observed. (Fig. 2E). However, the frequency of PD-L1+CD10− immature neutrophils in LDN in thyroid cancer patients was significantly higher than in healthy controls and this tendency was most noticeable in DTC and stages I–III. (Fig. 2F). Collectively, mature neutrophil and immature neutrophil proportion is associated tumor aggressiveness and PD-L1 positive immature neutrophil increased in advanced thyroid cancer.

### T-cell immunity-related gene signaling were reduced in thyroid cancer and associated with tumor type and advanced stage

**Fig. 3:**
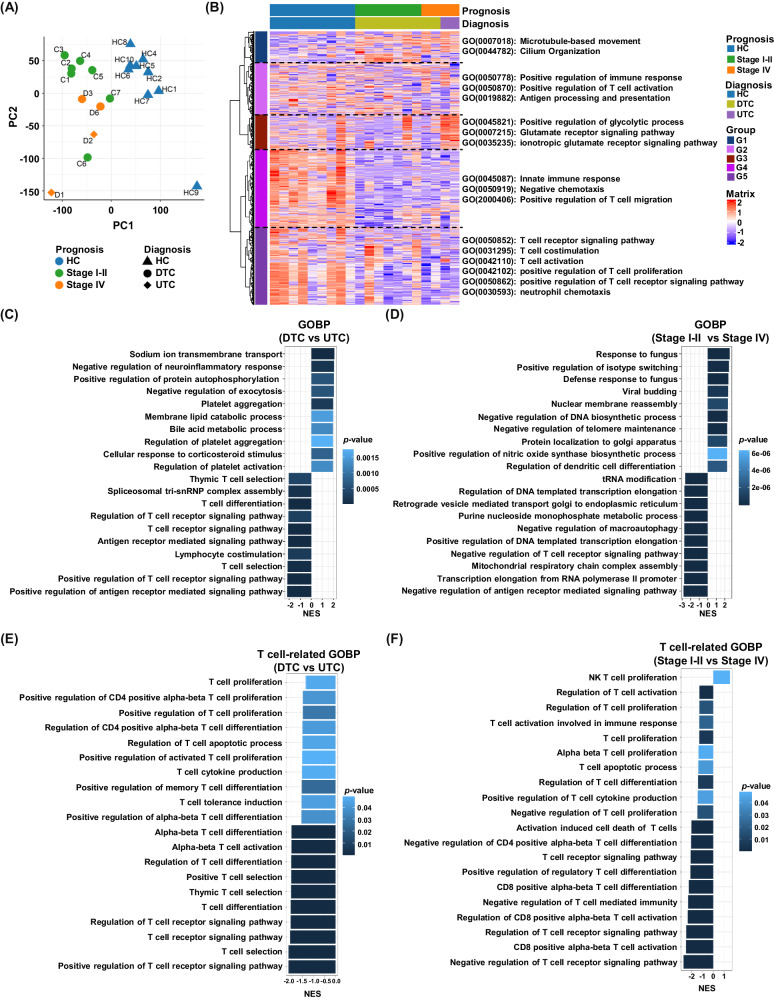
Transcriptome analysis of the neutrophils isolated from thyroid cancer and normal tissues. **A** Scatterplot of PC1 and PC2 from principal-component analysis (PCA) showing clusters in isolated neutrophils from whole blood of thyroid cancer patients (*n* = 11) and healthy controls (HC, *n* = 9). Color and shape of points indicate prognosis and diagnosis, respectively. **B** Heatmap showing hierarchical clustering and gene annotation of 2000 differentially expressed genes in neutrophils isolated from whole blood of thyroid cancer patients and healthy control volunteers. **C** Differentially enriched Gene Ontology Biological Process (GOBP) pathway from GSEA between isolated neutrophils from whole blood of DTC (*n* = 9) and UTC (*n* = 2). Horizontal axis and color represent normalized enrichment score (NES) and *p*-value, respectively. **D** Significantly enriched GOBP pathway between neutrophils from whole blood of Stage I–II (*n* = 7) and Stage IV (*n* = 4) patients. The horizontal axis and color represent NES and *p*-value, respectively. **E** Significantly enriched T-cell related Gene Ontology Biological Process (GOBP) pathway from GSEA between neutrophils obtained from whole blood of DTC (*n* = 9) and UTC (*n* = 2). The horizontal axis and color represent normalized enrichment score (NES) and *p*-value, respectively. **F** Significantly enriched T-cell related Gene Ontology Biological Process (GOBP) pathway from neutrophils obtained from whole blood of Stage I–II (*n* = 7) and Stage IV (*n* = 4) patients. The horizontal axis and color represent NES and *p* value, respectively.

Next, we performed transcriptome analysis of HDN obtained from whole blood from 11 thyroid cancer patients and 9 healthy control volunteers. The total amount of RNA from other participants was insufficient to create a library for sequencing. Principal component analysis (PCA) was performed to identify the expression patterns in each group (Fig. 3A). Color points were divided into groups based on prognosis, and the shape of the points indicated the divided groups based on diagnosis. We found that the tumor and healthy control (HC) groups exhibited different expression patterns. In addition, stages I–II and IV showed different expression patterns, which were similar in DTC and UTC (Fig. 3A). To identify differentially expressed genes (DEGs) and their gene ontology, hierarchical clustering using the top 2000 variable expressed genes. Heatmap analysis revealed distinct expression patterns in relation to tumor type or stage (Fig. 3B). We found that the gene signatures of group 3, such as the glycolytic process (GO0045821) and glutamate-related pathways (GO0007215 and GO0035235), were highly enriched in UTC patients. The gene signatures of group 4, such as innate immune response (GO0045087), negative chemotaxis (GO0050919), and positive regulation of T-cell migration (GO2000406), were significantly decreased in tumor neutrophils of whole blood compared to healthy controls. The gene signature of group 5 was composed of various T-cell-related pathways, such as T-cell activation (GO0042110), positive regulation of T-cell proliferation (GO0042102), and T-cell receptor signaling pathway (GO0050862), which showed a gradient change in relation to tumor type or tumor stage (Fig. 3B). These unsupervised analyses suggest the possibility of regulating the immune response via neutrophils in advanced cancer.

Next, we performed gene set enrichment analysis (GSEA) to identify significantly enriched pathways that contribute to the differences between normal and tumor tissues, as well as in divided groups based on tumor type and stage (Supplementary Fig. S2A). In the Gene Ontology Biological Process (GOBP) from GSEA, we found that chromatin- or protein refolding-related pathways were enriched, and lipoprotein-related pathways were downregulated in tumor samples (Supplementary Fig. S2B). To investigate the correlation of transcriptomic data with our investigations of enriched populations of immature neutrophils, we sorted T-cell immunity-related gene sets. Notably, we found that the gene scores of all significant gene sets related to T-cell immunity, including T-cell differentiation, activation, and proliferation, decreased in tumor samples, suggesting the impact of neutrophils on T-cell immunity in cancer (Supplementary Fig. 2C). To investigate the differences in gene signatures related to tumor type and stage, differential gene analysis was performed (Supplementary Fig. 2D, Supplementary Fig. 2E). In the unsupervised gene set analysis, we found similar results to the heatmap (Fig. 3B) when comparing DTC and UTC (Fig. 3C). T-cell immunity-related gene sets, such as T-cell differentiation, the T-cell receptor signaling pathway, and positive regulation of the T-cell receptor signaling pathway, were significantly decreased in patients with UTC (Fig. 3C). Stage IV tumors showed an upregulation of nuclear membrane reassembly and protein localization to the Golgi apparatus (Fig. 3D). Analysis focusing on gene sets related to T-cell immunity revealed that all T cell-related gene ontologies were significantly decreased in advanced cancer patients (Fig. 3E, F). These data suggest a relationship between HDN neutrophils and T-cell immunity in advanced thyroid cancer.

### Transcriptional profiling of neutrophils identified the tumor-specific genes in neutrophils related with tumor aggressiveness

**Fig. 4:**
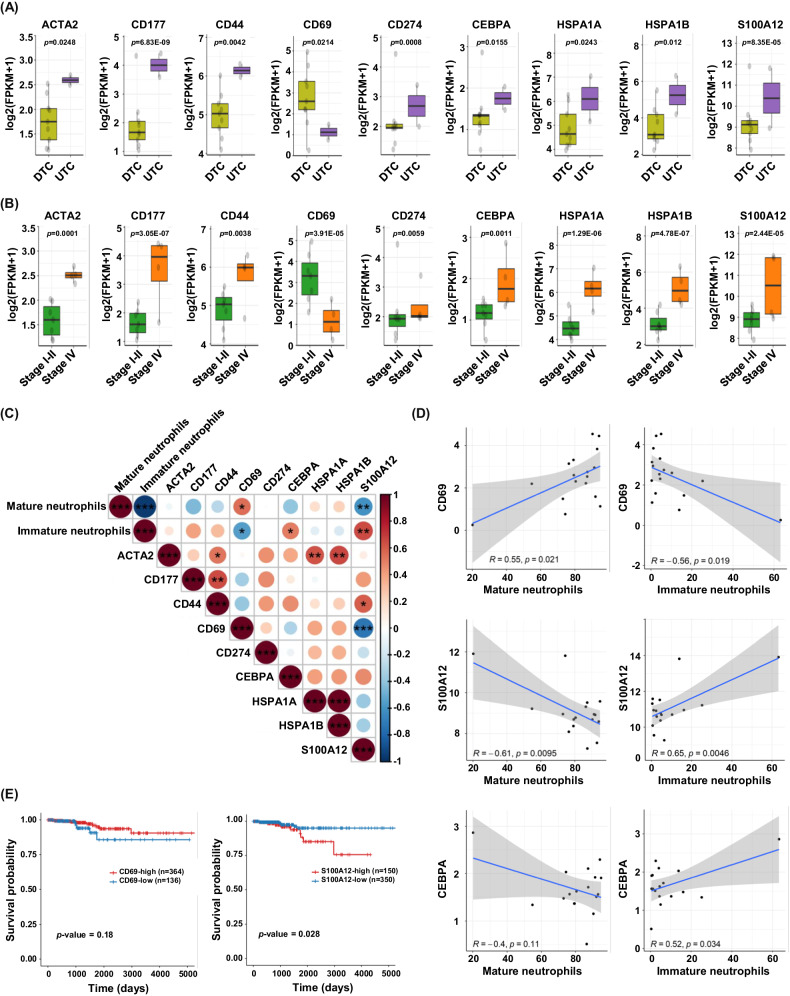
Genes in HDN neutrophils associated with tumor aggressiveness based on transcriptomics. **A** Comparison of neutrophil-related genes expression between neutrophils of DTC whole blood (*n* = 9) and neutrophils of UTC whole blood (*n* = 2). *p* value are written at the top of each plot. **B** Comparison of neutrophil-related genes expression in neutrophils obtained from whole blood of Stage I-II and Stage IV patients. *p* values are written at the top of each plot. **C** Correlation plot between neutrophil subsets and neutrophil-related genes in neutrophils obtained from whole blood of thyroid cancer patients (*n* = 11). Colors indicate Pearsons’s correlation coefficients. The corrplot R package was used. **D** Scatterplots of the correlation between immature neutrophils or mature neutrophils (horizontal axis) and log2 FPKM of neutrophil-related genes (vertical axis). (**E**) Validation of genes as predictive biomarker using TCGA database.

To identify tumor-specific genes related to tumor aggressiveness in neutrophils, we investigated genes related to neutrophil function. We obtained neutrophil-related genes according to Gene Ontology database annotations and searched the PubMed database. We found that several genes were highly ranked with significantly adjusted *p*-value and fold changes in the analyses (Supplementary Fig. 3A–C). After obtaining the neutrophil-related genes, we further analyzed the expression of signature genes in neutrophils obtained from whole blood of thyroid cancer patients, and then comparison of among cancer types and stages (Fig. 4).

ACTA2 (actin alpha 2, smooth muscle), a marker of neutrophil extracellular trap (NETs) [30], increased in advanced thyroid cancer (Fig. 4A, B). CD177, a marker of the proinflammatory neutrophil subset [31], and CD44, which plays an important role in neutrophil adhesion and migration [32], were upregulated both in UTC and advanced-stage thyroid cancer (Fig. 4A, B). The expression of CD69 was significantly downregulated, whereas expression of CD177, CD44, and CD274 (PD-L1) was significantly upregulated in both UTC and stage IV (Fig. 4A, B.). CEBPA, which is associated with the controlling of neutrophil development [33], was significantly upregulated in advanced cancer (Fig. 4A, B). We also investigated that increased some heat shock proteins (HSPs), HSPA1A and HSPA1B were upregulated in advanced cancer (Fig. 4A, B). We found that the expression of S100A12 was significantly associated with aggressive cancer (Fig. 4A, B). To validate the RNA-seq results, we identified several upregulated or downregulated genes using quantitative polymerase Chain Reaction (qPCR) in neutrophils from whole blood of same patients (Supplementary Fig. 3D). Most of the genes showed the same trend as transcriptomics, and in particular, CD177, HSPA1A, HSP1B, and S100A12 were significantly increased in advanced thyroid cancer compared to in indolent thyroid cancer (Supplementary Fig. 3D.). Using a correlation plot, we investigated the expression of candidate genes and proportion of mature and immature neutrophils (Fig. 4C). Among candidate genes, CD69 was significantly positively correlated with mature neutrophils and negatively correlated with immature neutrophils. Immature neutrophils negatively correlated with CD69 (Fig. 4D). In contrast, S100A positively correlated with immature neutrophils and negatively correlated with mature neutrophils (Fig. 4D). These results suggest that those genes are promising neutrophil markers for distinguishing advanced cancers. To explore the potentiality of predictive biomarker, we investigated the relationship between candidate genes and survival using the TCGA database and found that S100A12 expression was related to worse survival (Fig. 4E). Thus, we explored tumor-specific genes in neutrophils and determined the impact of genes related to the prognosis of thyroid cancer.

### Evaluation of difference of T-cell subsets in relation to tumor aggressiveness

**Fig. 5:**
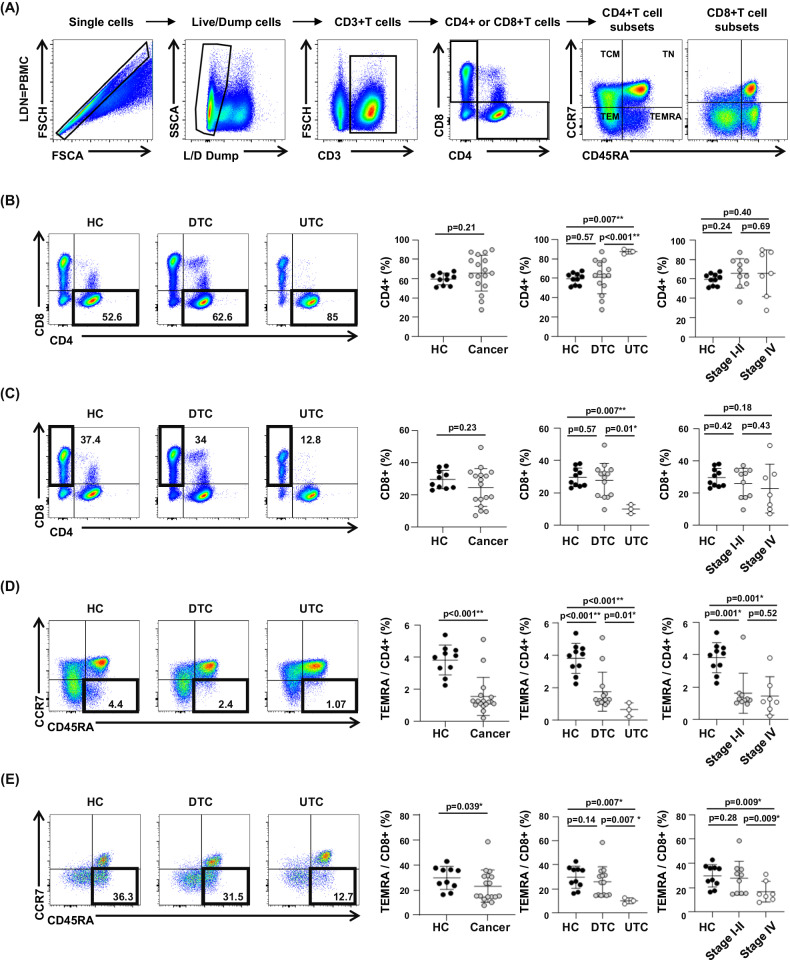
Flow cytometry analysis of lymphocyte subsets in thyroid cancer and healthy controls. **A** Gating strategies of lymphocytes applied in flow cytometry analyses. **B** Frequency of CD4+ T cells in patients with cancer and healthy controls. **C** Frequency of CD8+ T cells in cancer patients and healthy controls. **D** Frequency of effector memory cells re-expressing CD45RA (TEMRA) CD4+ T cells in patients with cancer and healthy controls. **E** Frequency of TEMRA CD8+ T cells in patients with cancer and healthy controls. Data were expressed as the mean ± SD (HC, *n* = 10; Cancer, *n* = 17; DTC, *n* = 14; UTC, *n* = 3; Stage I–II, *n* = 10; Stage IV, *n* = 7).

The proportions of T-cell subsets were identified in the same patients to identify the link between T-cell cell immunity and neutrophil-related gene signatures using flow cytometry (Fig. 5A). Although there was no significant difference between healthy controls and patients with cancer, the frequency of CD4+ T cells was significantly higher in patients with UTC compared to in DTC and healthy controls (Fig. 5B). In contrast, the frequency of CD8+ T cells was significantly lower in patients with UTC than in those with DTC and in healthy controls (Fig. 5C). Next, we compared the frequency of effector memory cells re-expressing CD45RA (TEMRA). Participants with cancer had significantly smaller populations of TEMRA among the CD4+ and CD8+ T cells than healthy controls (Fig. 5D, E). Moreover, the frequency of TEMRA CD4+ T cells and CD8+ T cells was significantly lower in patients with UTC than in those with DTC (Fig. 5D, E). Although there is no change of cell numbers of TEMRA CD4+ T cells between Stage I-II and Stage IV patients, TEMRA CD8+ T cells were significantly decreased in stage IV patients. Collectively, we observed decrease of TEMRA CD4+ and CD8+ T-cell subsets in advanced cancer, similar to our transcriptomic data.

### Evaluation of neutrophil and T-cell gene proportions and neutrophil-related genes associated with tumor type using bulk RNA sequencing

**Fig. 6:**
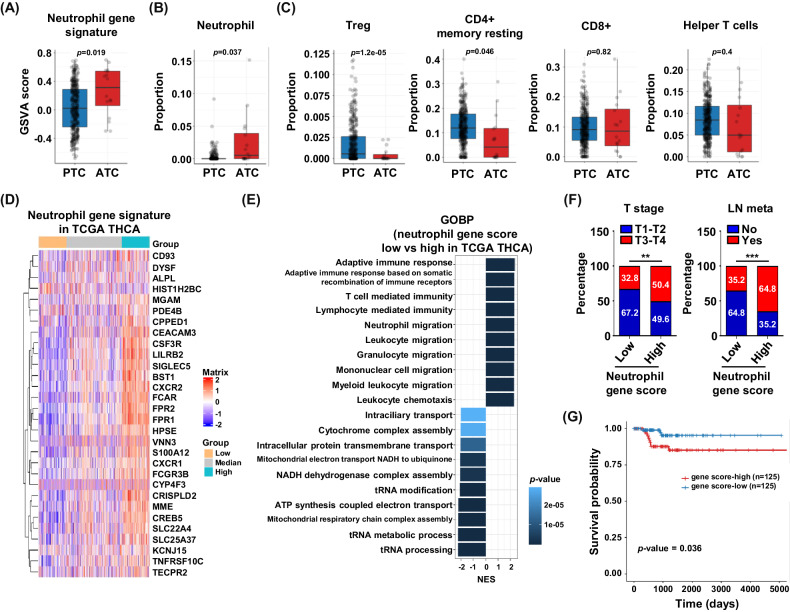
Importance of neutrophil in thyroid tumor using bulk RNA sequencing. **A** Comparison of neutrophil gene signature between papillary thyroid cancer (PTC, *n* = 348) and anaplastic thyroid cancer (ATC, *n* = 16) in our bulk RNA-seq analysis (GSE213647). *p* value are written at the top of bar plot. Comparison of neutrophil proportion (**B**) and T-related immune cells proportion (**C**) between PTC and ATC using bulk RNA sequencing by deconvolution analysis. *p*-values are written at the top of each plot. Significantly enriched neutrophil- (**D**) and T cell-related GOBP (**E**) from comparison in PTC and ATC. The horizontal axis and color represent normalized enrichment score (NES) and *p*-value, respectively. **D** Heatmap showing neutrophil gene signature in THCA (*n* = 500) of TCGA database. **E** Enriched GOBP in neutrophil gene signature -high and -low group. Horizontal axis and color represent normalized enrichment score (NES) and *p*-value, respectively. **F** Association of T cell and Lymph node metastasis between high neutrophil tumor and low neutrophil tumor. **G** Survival probability in disease-free according to neutrophil gene signature expression. Data were expressed as the mean ± SD.

**Fig. 7:**
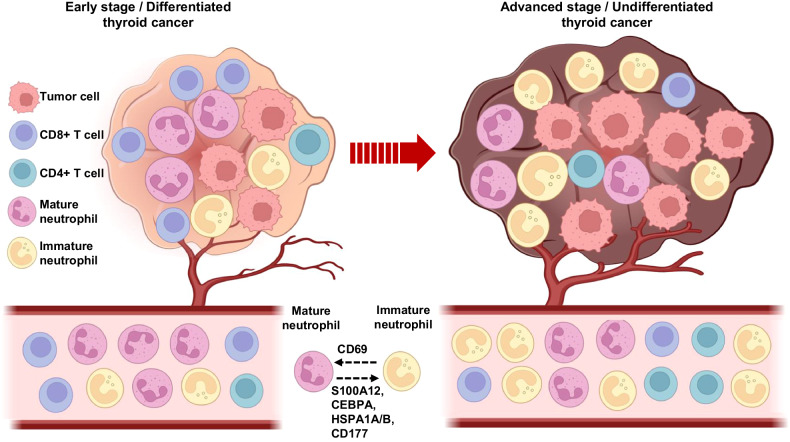
The proposed schematic representation of neutrophil diversity in advanced thyroid cancer. The proportions of tumor infiltrated neutrophils were increased and related with poor prognosis in advanced thyroid cancer. Moreover, the proportion of circulating immature neutrophils were significantly increased and the proportions of circulating CD8+ T effector cells were significantly decreased in advanced stage thyroid cancer patients compared to early stage thyroid patients. Downregulation of CD69 and upregulation of S100A12, CEBPA, HSPA1A, HSPA1B, and CD177 were associated with increased immature neutrophil in thyroid cancer.

To validate the importance of neutrophils in advanced thyroid cancer, we explored the proportions of neutrophils and other immune cells via deconvolution analysis of bulk RNA sequencing data. We used our bulk RNA sequencing data of thyroid cancer tissues from previous study [34] to identify the impact of neutrophil gene signature expression and immune cells proportion. The neutrophil gene signature score was calculated using the GSVA package with neutrophil gene lists [13]. The neutrophil gene signature was significantly higher in ATC (anaplastic thyroid cancer) than in PTC (papillary thyroid cancer) (Fig. 6A). We analyzed tumor-infiltrating immune cells and neutrophils using bulk RNA-seq results via the TIMER2.0 webtool for deconvolution. Similarly, the proportion of neutrophils was significantly higher in ATC group than in PTC group (Fig. 6B). Regulatory T Cells and CD4+memory T cells were decreased in ATC compared to PTC (Fig. 6C), although there was no significant difference in other T cells, including CD8+ and helper T cells. Next, we validated the impact of neutrophils on tumor aggressiveness in a large cohort and analyzed neutrophil-related gene scores in TCGA database (Fig. 6D). Based on the neutrophil gene score, we divided patients with PTC from TCGA into high- and low-neutrophil groups. The high neutrophil score group revealed upregulation of several neutrophil-related pathways, such as neutrophil migration and leukocyte migration, and downregulation of tRNA metabolic process, NADH dehydrogenase complex assembly, and ATP synthesis coupled electron transport signaling (Fig. 6E). Advanced stage (T stage 3–4) and LN metastasis significantly increased in the high neutrophil group (Fig. 6F), and recurrence-free survival was significantly worse in the high neutrophil group than in the low neutrophil group (Fig. 6G). Collectively, our data suggest that the neutrophil population is significantly linked to tumor aggressiveness in advanced thyroid cancer using bulk RNA sequencing data (Fig. 7).

## Discussion

Early reports suggest that during tumor progression, high-density mature neutrophils (HDNs) are converted to low-density neutrophils (LDNs) with loss of anti-tumor properties due to TGF beta signaling [35, 36]. However, recent studies based on advanced technologies, such as single-cell transcriptomics, have identified that not only the distinction between LDN and HDN, but also the proportion and molecular pathways of precursor neutrophils and immature and mature neutrophils are important in disease status [37–39]. In a healthy state, mature neutrophils are predominantly detected in the circulation, whereas in a disease state, immature neutrophils are released into the circulation due to various inflammatory stimuli [40]. Furthermore, while mature neutrophils expert protective effects against neutrophil diseases, immature neutrophils are non-proliferative and act as a reservoir that is quickly deployed into circulation [2]. The heterogeneity of various HDNs, LDNs, immature neutrophils, and mature neutrophils in diseases such as sepsis, inflammation, and cancer has been reported; however, but there are no direct studies on thyroid cancer. Our study dissected the neutrophil heterogeneity by FACS analysis of circulating neutrophils using fresh blood samples from patients with thyroid cancer. HDN accounts for a larger proportion of CD45+ positive cells than LDN. The overall population of LDN, which was suggested to increase in disease exacerbation by stimulus, did not differ between advanced cancer and healthy control. In contrast, HDN proportion were significantly decreased in both UTC and Stage IV patients. Notably, when we divided these HDNs and LDNs into mature and immature neutrophils based on the presence or absence of CD10 expression, we observed a significant increase in immature neutrophils and a decrease in mature neutrophils in HDN, especially in advanced cancer patients. Similar to previous results of neutrophils about the immunosuppressive cellular network through defective T- and NK-cell cytotoxicity [41, 42], we also found that immature neutrophils expressing PD-L1, an immunoescape signaling-related marker known to reduce the activity of cytotoxic T cells, increased compared with healthy controls in both HDN and LDN. These results suggest that HDN neutrophils, which account for the largest proportion of circulating neutrophils, might have pivot role in advanced thyroid cancer via immunoescape signaling.

Recently, cancer immunotherapy targeting cytotoxic T cells and immunoescape signaling of tumor cells has changed the direction of cancer treatment; however, some patients still experience serious immunological side effects and poor treatment response, and many studies have focused on ways to overcome the irreversible dysfunction of cytotoxic CD8 T cell activity [43–45]. The link between neutrophils and T cells and their properties in the tumor microenvironment suppress antitumor T-cell immunity through reactive oxygen species (ROS) production [46], but neutrophil-T-cell interactions are not fully understood. Recently, neutrophils with high glycolytic capacity have been shown to accumulat in the spleen and inhibit T-cell activity by stealing glucose from the surrounding T cells during the progression of breast cancer [47]. Using heatmap analysis, we found that peripheral neutrophils in ATC represented the upregulation of glycolytic process, glutamate receptor signaling pathway. Other metabolic pathways, such as lipid metabolism and corticosteroid metabolic pathways were significantly upregulated in advanced thyroid cancer along with downregulation of various T-cell-related signaling pathways. Although we could not directly identify the link of neutrophils with T-cell immunity, we observed the altered metabolic pathways and T-cell-related signaling in circulating neutrophils from patients with thyroid cancer. A previous study based on rodent ATC models showed that the presence of PMN-MDSC, which are the neutrophil precursors, was related to ATC treatment failure [48]. They found that lenvatinib monotherapy, one of the standard treatments for ATC, induces an increase in circulating myeloid-derived suppressor cells (MDSCs). Moreover, combination therapy with anti-Gr-1, which inhibits the proliferation of MDSCs, was more effective in regulating tumor growth. However, the impact of neutrophils on T-cell immunity in advanced thyroid cancer has not been fully elucidated. In our transcriptomic data, T-cell-related pathways, such as T-cell activation, T-cell proliferation, and T-cell differentiation were significantly decreased in neutrophils from both UTC and stage IV cancer. These results were validate using FACS analysis. Therefore, we identified the involvement of neutrophils and T cells in the tumor aggressiveness of thyroid cancer.

C/EBPα and C/EBPβ are transcription factors during neutrophil development and stimulated by diverse inflammatory cytokines [37, 49]. In our data, C/EBPα gene expressions were upregulated in advanced cancer and were significantly positively correlated with immature neutrophils in HDN. Although we could not identify the exact role of C/EBPα in thyroid cancer, it might be related with tumor induced stimulatory cytokines robust the increase of neutrophil differentiation. We also observed that CD69, one of the costimulatory factor for T-cell activation [50, 51], decreased in UTC and stage IV patients, along with the downregulation of T-cell metabolic pathway. Similarly with previous study that revealed that activated neutrophils enhance their lysozyme release by crosslinking of CD69 [52], our data revealed that CD69 expression in neutrophils is associated with immunosuppressive phenotype in thyroid cancer. We also found an association between S100A12 in neutrophils and neutrophil heterogeneity and validated the role of S100A12 as a predictive biomarker using TCGA data. Recently, S100A12-mediated inflammatory processes have been observed in various disease conditions [53–55]. Our results are constituent with previous study showing that S100A12 expression is upregulated in tumors and that regulation of it induced decreased tumorigenesis [56].

Previously, a large pan-cancer meta-analysis confirmed that neutrophil features are associated with poor prognosis [57], however, but there are no reports on their effect in undifferentiated thyroid cancer. In this study, through deconvolution analysis of bulk RNA sequences, we showed in human data that neutrophil populations were indeed larger in ATC than in PTC. Furthermore, TCGA data were validated to verify the importance of PBMC-based research by identifying the correlation with tumor aggressiveness, such as recurrence, T stage, and lymph node metastasis, in high and low neutrophil tumors. These results suggest the importance of the heterogeneity of circulating neutrophils not only at the circulating level, but also their association with tissue-based infiltrating neutrophils and the impact of neutrophils in the tumor microenvironment.

Our data revealed an association of the immature-to-mature neutrophil ratio and the differentiation status of the disease, however, we could not provide functional data to confirm this assumption and the sample size is small. Further large-scale functional study is necessary to identify the role of immature-to mature neutrophil heterogeneity in advanced thyroid cancer.

Collectively, we found that neutrophil heterogeneity in thyroid cancer not only unravels critical insights into the intricate interplay between neutrophils and T-cell immunity but also sheds light on its potential clinical implications. The prevalence of circulating immature high-density neutrophils and their immunosuppressive features in undifferentiated thyroid cancers suggest the importance of understanding neutrophil dynamics in the context of tumor progression in thyroid cancer.

## Materials and methods

### Study population

Blood samples were collected from 10 healthy volunteers and 17 patients with thyroid cancer. FACS analysis was performed on a total of 27 participants, and transcriptomics from HDN isolated from 20 participants was analyzed (Fig. 1A). For analyzing FACS analysis, 2 patients with anaplastic thyroid cancer and 1 patient with poorly differentiated cancer were defined as a group called UTC, and 2 patients with ATC were included in transcriptomics analysis (Supplementary Data 1). To validate the role of neutrophil in thyroid cancer, we used transcriptomics data from thyroid cancer tissue that we used in our previous study [34]. All samples were treatment- naïve patients. Informed consent was obtained from all the participants. In this study, maximum tumor size was not utilized for enrollment criteria. In the case of undifferentiated cancer, debulking surgery or total thyroidectomy is the principle of treatment regardless of the size of the tumor.

### Peripheral blood mononuclear cells (PBMCs) and neutrophil isolation

Peripheral blood mononuclear cells (PBMCs) and neutrophils were isolated from whole blood by standard Ficoll-Paque (GE Healthcare) density gradient centrifugation immediately after blood collection within 10 minutes. The interphase layer resulting from the gradient was diluted and washed twice with Dulbecco’s phosphate-buffered saline (PBS) to obtain PBMCs and low-density neutrophils (LDNs) from the top and high-density neutrophils (HDNs) from the bottom. The pellet obtained after centrifugation of peripheral blood on Ficoll, containing erythrocytes and high-density neutrophils (HDNs), was subjected to hypotonic lysis (155 mM NH_4_Cl, 10 mM KHCO_3_, 0.1 mM EDTA, pH 7.4) on ice for 15 min. Following red cell lysis, the granulocytes were recovered by centrifugation, washed with MACS rinsing buffer, and then resuspended in 50 ml MACS running buffer. HDNs were isolated by magnetic cell sorting using CD16 microbeads, according to the manufacturer’s instructions. Cell viability and quality were evaluated by the Cellometer Auto 2000 system (Nexcelom Bioscience LLC) using Acridine Orange (AO)/PI staining, and after confirming that the viability of cells was over 95%, the next experiment was performed.

### Flow cytometry analysis

Multicolor flow cytometry of peripheral blood cells was performed at Chungnam National University Hospital (CNUH). To exclude dead cells, single-cell suspensions were incubated for 20 min with a viability dye (LIVE/DEAD Fixable Aqua, Thermo Fisher). For neutrophil analysis, cells were stained with fluorochrome-conjugated antibodies, including anti-CD66b (Brilliant Violet 421; BD Biosciences), anti-CD56 (Brilliant Violet 510; BD Biosciences), anti-CD3, anti-CD19, anti-CD20 (Brilliant Violet 510; BioLegend), anti-PD-L1 (Brilliant Violet 711; BioLegend), anti-CD45 (Brilliant Violet 786; BioLegend), anti-CD10 (VioBright FITC; BioLegend), and anti-CD16 (APC-Cy7; BioLegend). For T-cell analysis, the cells were stained with fluorochrome-conjugated antibodies, including anti-CD14, anti-CD19 (Brilliant Violet 510; BD Biosciences), anti-CD4 (Brilliant Violet 876; BD Biosciences), anti-CD45RA (APC-R700; BD Biosciences), anti-CD3 (APC-Cy7; BD Biosciences), anti-CD8 (VioBright FITC; BioLegend), and anti-CCR7 (PerCP-Cy5.5; BioLegend). Flow cytometry was performed using a BD LSR Fortessa X-20 flow cytometer (BD Biosciences), and the data were analyzed using FlowJo software (Tree Star, Ashland, OR, USA). When gating subpopulations of cells, the distinction between positive and negative was based on Fluorescence Minus One (FMO) control.

### RNA extraction for sequencing

Total RNA was extracted from isolated neutrophils from whole blood of patients and healthy volunteer using the TRIzol Reagent (Invitrogen, Carlsbad, CA, USA). The quality of the extracted total RNA was evaluated using an Agilent 2100 Bioanalyzer RNA Nano Chip (Agilent Technologies, Santa Clara, CA, USA). The extracted RNA was used to construct RNA libraries using the SMART-Seq v4 Ultra Low Input RNA Kit for Sequencing (Takara Bio USA, Mountain View, CA, USA) or TruSeq RNA Library Preparation v2 Kit (Illumina, San Diego, CA, USA), according to the manufacturer’s instructions. Library quality was analyzed using an Agilent 2100 Bioanalyzer and Agilent DNA 1000 kit (Agilent). All samples were sequenced on a HiSeq X Ten system (Illumina), yielding an average of 38 million paired-end 100 nucleotide reads. The reads were mapped to the human reference sequence (GRCh38) using HISAT2 (v2.1.0) to produce aligned reads. Transcription assembly was performed through the StringTie (v2.1.3 b) program using the information of the aligned reads based on reference. The expression profile obtained by transcript quantification of each sample was extracted with fragments per kilobase per million mapped fragments (FPKM), reads per kilobase per million mapped reads (RPKM), and transcripts per million (TPM), which is the normalization value considering read count, transcript length, and depth of coverage.

### RNA sequencing analysis

To observe differential gene expression in relation to diagnosis or prognosis, we performed hierarchical cluster analysis using the ComplexHeatmap R package with log10 fragments per million mapped fragments (FPM). Clusters within the heatmap were annotated by Gene Ontology (GO) terms from the Database for Annotation, Visualization, and Integrated Discovery (DAVID). Additionally, we identified DEGs by dividing them into two groups based on prognosis or diagnosis. Differential expression analysis (DEA) was performed using the DESeq2 R package and enrichment analysis was performed using the fgsea R package. To analyze the relationship between neutrophil subsets and neutrophil-related genes, we performed a corrplot R package using Pearson’s correlation. The neutrophil gene signature score was calculated using the GSVA package and neutrophil gene signature list (reference PMID 27737879). To analyze the proportion of immune cells in the bulk RNA-seq, we used TIMER2.0 (http://timer.cistrome.org/) [58].

### Quantitative PCR

Complement DNA (cDNA) was synthesized by M-MLV reverse transcriptase (Invitrogen), oligo-dT primers (Promega, Madison, WA, USA), and extracted RNA. Specific sequences were amplified from each cDNA sample using a SYBR Green PR Master Mix (Applied Biosystems, Foster City, CA, USA) and the primers sequences listed in Supplementary Data. 5. Relative expression was calculated by normalizing against 18 S mRNA expression on a 7500 Real-Time PCR System Software (v2.0.6, Applied Biosystems).

### Analysis of The Cancer Genome Atlas (TCGA) database

We obtained genomic data for papillary thyroid carcinoma (THCA) from TCGA data portal (http://tcga-data.nci.nih.gov). Gene expression data generated via RNA sequencing and clinical parameters in patients with papillary thyroid carcinoma (*n* = 500), as well as overall survival and disease-free survival, were analyzed. We analyzed the survival probability of the two groups based on the values presented in the Human Protein Atlas (https://www.proteinatlas.org/).

### Statistical analysis

Continuous variables are presented as mean ± standard deviation or standard error, and categorical variables are presented as numbers and percentages. Unpaired Student’s t-tests or Mann–Whitney *U* tests were used to compare continuous variable means, while the Chi-square or Fisher’s exact test was used to compare categorical variable distributions. Statistical significance for all analyses was established with a two-tailed *p* value < 0.05. Correlations were calculated using Pearson’s correlation (two-tailed *p* value) in the R program. Statistical analyses and graphs were generated using SPSS Version 26.0. (IBM corp., Armonk, NY, USA), R, GraphPad Prism 9.4.1. software (GraphPad Software Inc., San Diego, CA, USA) and OriginPro 2021 (OriginLab Corp., Northampton, MA, USA).

### Supplementary information

supplementary data

supplementary figure 1

supplementary figure 2

supplementary figure 3

## Data Availability

The bulk RNA sequencing data for this study were deposited in the GEO database under accession number GSE239976.
