# Supplementary figures and images for: Neutrophil diversity is associated with T-cell immunity and clinical relevance in patients with thyroid cancer
Source: Cell Death Discov. 2024 May 8;10:222. doi: 10.1038/s41420-024-01970-z (PMC11078953; doi:10.1038/s41420-024-01970-z)

Supplementary Figure 1.

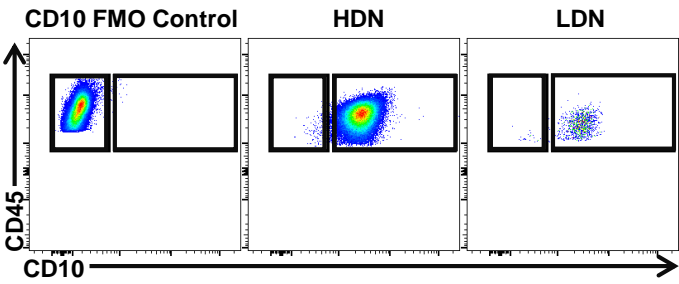

Supplement: Supplementary file 2 — supplementary figure 1 [file 41420_2024_1970_MOESM2_ESM.pdf]

(A)

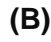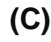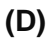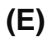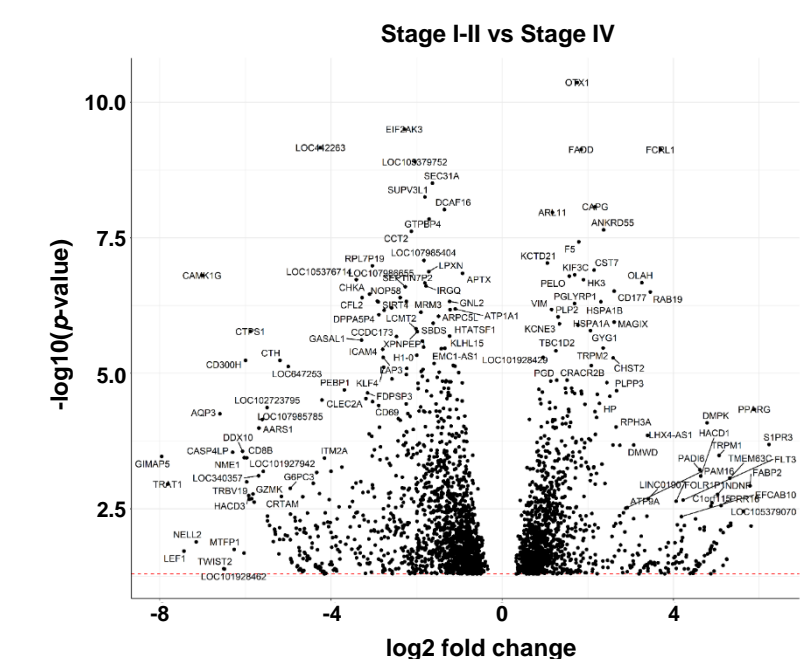

Supplement: Supplementary file 3 — supplementary figure 2 [file 41420_2024_1970_MOESM3_ESM.pdf]

Supplementary Figure 3.

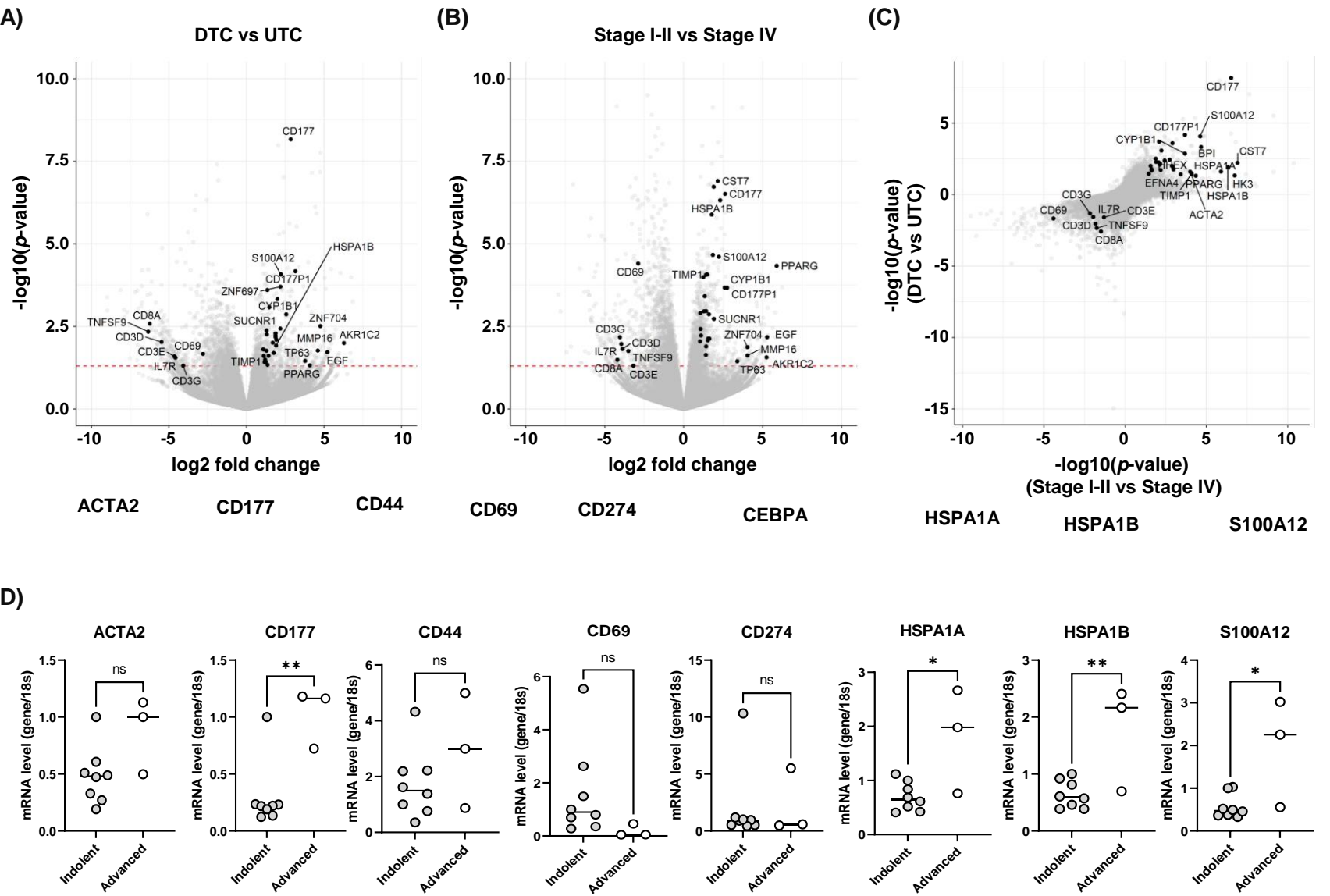

Supplement: Supplementary file 4 — supplementary figure 3 [file 41420_2024_1970_MOESM4_ESM.pdf]
